# Contemporary preclinical mouse models for pediatric rhabdomyosarcoma: from bedside to bench to bedside

**DOI:** 10.3389/fonc.2024.1333129

**Published:** 2024-02-02

**Authors:** Illya Martynov, Lajwanti Dhaka, Benedikt Wilke, Paul Hoyer, M. Reza Vahdad, Guido Seitz

**Affiliations:** ^1^ Department of Pediatric Surgery and Urology, University Hospital Giessen-Marburg, Marburg, Germany; ^2^ Department of Pediatric Surgery, University Hospital Giessen-Marburg, Giessen, Germany

**Keywords:** rhabdomyosarcoma, *in vivo*, mouse model, translational impact, RMS

## Abstract

**Background:**

Rhabdomyosarcoma (RMS) is the most common pediatric soft-tissue malignancy, characterized by high clinicalopathological and molecular heterogeneity. Preclinical *in vivo* models are essential for advancing our understanding of RMS oncobiology and developing novel treatment strategies. However, the diversity of scholarly data on preclinical RMS studies may challenge scientists and clinicians. Hence, we performed a systematic literature survey of contemporary RMS mouse models to characterize their phenotypes and assess their translational relevance.

**Methods:**

We identified papers published between 01/07/2018 and 01/07/2023 by searching PubMed and Web of Science databases.

**Results:**

Out of 713 records screened, 118 studies (26.9%) were included in the qualitative synthesis. Cell line-derived xenografts (CDX) were the most commonly utilized (n = 75, 63.6%), followed by patient-derived xenografts (PDX) and syngeneic models, each accounting for 11.9% (n = 14), and genetically engineered mouse models (GEMM) (n = 7, 5.9%). Combinations of different model categories were reported in 5.9% (n = 7) of studies. One study employed a virus-induced RMS model. Overall, 40.0% (n = 30) of the studies utilizing CDX models established alveolar RMS (aRMS), while 38.7% (n = 29) were embryonal phenotypes (eRMS). There were 20.0% (n = 15) of studies that involved a combination of both aRMS and eRMS subtypes. In one study (1.3%), the RMS phenotype was spindle cell/sclerosing. Subcutaneous xenografts (n = 66, 55.9%) were more frequently used compared to orthotopic models (n = 29, 24.6%). Notably, none of the employed cell lines were derived from primary untreated tumors. Only a minority of studies investigated disseminated RMS phenotypes (n = 16, 13.6%). The utilization areas of RMS models included testing drugs (n = 64, 54.2%), studying tumorigenesis (n = 56, 47.5%), tumor modeling (n = 19, 16.1%), imaging (n = 9, 7.6%), radiotherapy (n = 6, 5.1%), long-term effects related to radiotherapy (n = 3, 2.5%), and investigating biomarkers (n = 1, 0.8%). Notably, no preclinical studies focused on surgery.

**Conclusions:**

This up-to-date review highlights the need for mouse models with dissemination phenotypes and cell lines from primary untreated tumors. Furthermore, efforts should be directed towards underexplored areas such as surgery, radiotherapy, and biomarkers.

## Introduction

Rhabdomyosarcoma (RMS) is a highly aggressive soft tissue sarcoma that develops from primitive mesenchymal cells and exhibits features of skeletal muscle. RMS primarily affects children, comprising approximately 3-4% of all childhood cancers (1). Due to its mesenchymal origin, RMS can develop in various locations throughout the body. Based on the data of the Children’s Oncology Group (COG) and the European Pediatric Soft Tissue Sarcoma Study Group (EPSSG), the most common anatomic locations in pediatric rhabdomyosarcoma are paratesticular (20%), parameningeal (20%), retroperitoneum/peritoneum/trunk (16%), extremities (14%), head/neck (9%), and bladder/prostate (7-8%) (2). In metastatic RMS, which accounts for 15-20% of cases, the most commonly affected sites, including multiple-site metastases, are the lungs (66.2%), bone marrow (63.7%), and bones (50.0%) (3). Historically, RMS is characterized by four distinct histologic subtypes: embryonal (eRMS), alveolar (aRMS), pleomorphic (plRMS), and spindle cell/sclerosing (ssRMS). eRMS and aRMS represent the two main subgroups, comprising 38.8% and 22.3% of all cases, respectively (4).

Currently, the only genomic marker incorporated in risk stratification is the *PAX-FOXO*1 fusion status (2). In general, fusion-positive (FP-RMS) tumors are more aggressive, with a higher potential for dissemination and disease recurrence compared to fusion-negative RMS (FN-RMS) (5). However, the genetic profiles within FP-RMS and FN-RMS also differ. FP-RMS is mostly associated with *CDK4* (13%), *MYCN* (10%), *BCOR* (6%), *NF1* (4%), and *TP53* (4%) mutations, whereas in FN-RMS, mutations in *RAS* pathway members are the most common, affecting up to 32% of patients, including *NRAS* (17%), *KRAS* (9%), and *HRAS* (8%). Among FP-RMS, *MYCN* overexpression and *TP53* alteration are associated with more aggressive disease. In FN-RMS, *MYOD1* and *TP53* serve as indicators for a poor prognosis (2). It has also been shown that tumor biology and clinical behavior of FN-aRMS closely resemble eRMS, whereas they significantly differ from PF-aRMS (6). This indicates that the molecular status rather than the histological subtype is of prognostic significance (2, 7).

The management of RMS is multimodal, including chemotherapy, surgical resection, and/or radiation therapy (8). Treatment intensity depends on risk stratification (9, 10). Children with low-risk RMS, treated with frontline multi-modal therapy, attain a 90% relapse-free survival rate, whereas those with high-risk phenotypes (aRMS/FP-RMS), metastatic or relapsed RMS, experience a poor overall cure rate, usually below 30% (11, 12).

Preclinical *in vivo* models play a crucial role in advancing the understanding of RMS biology and testing novel diagnostic methods and therapies (13). The ideal RMS model should accurately recapitulate the biological and molecular features of human RMS, and exhibit similar tumor growth dynamics and metastatic behavior. Since meeting all these requirements is not possible in the ‘real’ world, it is crucial to select the most appropriate model based on specific inquiries and to critically interpret the data in the context of the inherent limitations of the models.

This systematic survey aimed to critically assess the contemporary literature on preclinical mouse models for RMS, enabling the characterization of tumor phenotypes (bedside-to-bench modeling) and providing an overview of model utilization areas (bedside-to-bench).

## Methods

We did an electronic search of PubMed and Web of Science from 01/07/2018 to 15/07/2023. The following terms were used: “animal” OR “animal model” OR “preclinical studies” OR “preclinical study” OR “experimental animals” OR “experimental animal” OR “laboratory animal” OR “laboratory animals” OR “rodents” OR “murine” OR “animal disease model” OR “mice” OR “mouse” AND “rhabdomyosarcoma”. Published studies on mouse models for RMS, regardless of the intended purpose of model utilization, were included. Case studies, cross-over studies, review articles, editorials, commentaries, and letters were excluded. Additionally, other murine (e.g., rats) and non-murine models (e.g., zebrafish) as well as exclusively *in vitro*, ex vivo, or in ovo studies were excluded. Studies included in the qualitative synthesis were accessed for the following parameters: first author, year of publication, country (according to first author), study category (basic/translational research), aim of the study (tumorigenesis, disease modeling, drug testing/treatment, imaging, radiation, long-term effects, biomarker), mouse model (syngeneic allograft, cell line-derived xenograft (CDX), patient-derived xenografts (PDX), genetically engineered mouse models (GEMM), number of animals (overall number per study and number per treatment group), histological RMS subtype (aRMS/FP-RMS, eRMS/FN RMS), mouse background, cell lines (human/murine, aRMS, eRMS, plRMS, ssRMS), engraftment site [ectopic, orthotopic, intravenous (i.v.), intraperitoneal (i.p.)], metastases (yes/no), and metastases type (spontaneous, experimental via intravenous or intraperitoneal cell application)). Ectopic mouse models were defined as those in which the transplantation or introduction of cells or tissues occurred at a location outside their natural site within the organism, specifically into the subcutaneous tissue of the mouse. Orthotopic model systems were defined as those in which cells or tissues were transplanted or introduced into the musculature, thus imitating the clinical phenotype of extremity RMS. We used the SYRCLE Risk of Bias - assessment tool for animal studies to estimate the risk of bias (RoB) (14). A ‘yes’ score indicates a low risk of bias; a ‘no’ score indicates a high risk of bias; and an ‘unclear’ score indicates an unknown risk of bias. We judged the quality of the included papers using the following items: 1) sequence generation (selection bias), 2) baseline characteristics (selection bias), 3) allocation concealment (selection bias), 4) random housing (performance bias), 5) blinding (performance bias), 6) random outcome assessment (detection bias), 7) blinding for outcome (detection bias). The statement of conflicts of interest was not considered in the study quality assessment. We developed a semi-quantitative scoring system to assess the grade of tumor reproducibility (bedside-to-bench-score) and the potential translationality (bench-to-bedside-score), which were based on core experimental variables, including animal system category, applied cell lines, and engraftment sites. The detailed characteristics of the scoring systems are summarized in **Tables 1**, **2**. For bedside-to-bench-scoring, studies focused on basic research were excluded, as their *a priori* goals may not have immediate practical applications. Briefly, Bedside-to-bench-score: low (syngeneic models), moderate (ectopic CDX models), high (orthotopic CDX or GEMM), and very high (ectopic or orthotopic PDX models). Bench-to-bedside scoring: low (no translational impact, e.g., new drug testing on ectopic CDX), moderate (distant translation possible, e.g., establishment of PDX tumor bank), high (direct translational impact, e.g., MRI/histology-correlation studies, studies on long-term effects after radiotherapy), very high (immediate translational impact by using complex mouse systems, e.g., Single Mouse Trials). The category assignment for the ‘bench-to-bedside’ score was determined using five questions, each scored from “+” to “++++”: Q1: “How applicable are the study findings to real-world clinical scenarios?”, Q2: “Does the study directly address clinical questions or problems?”, Q3: “How likely are the study findings to be translated into clinical applications?”, Q4: “Does the study offer practical solutions or tools for clinicians?”, Q5: “How likely are the findings to be incorporated into clinical practice?”. These resulted in four bench-to-bedside score categories: low (no translational impact, e.g., new drug testing on ectopic CDX), moderate (distant translation possible, e.g., establishment of PDX tumor bank), high (direct translational impact, e.g., MRI/histology-correlation studies, studies on long-term effects after radiotherapy), very high (immediate translational impact by using complex mouse systems, e.g., Single Mouse Trials). All descriptive statistics were done using Jamovi (Version 2.3.16) and GraphPad Prism (Version 10.0.3) software. The choropleth map was created using Datawrapper (https://www.datawrapper.de).

**Table 1:** “Bedside-to-bench” scoring system.

Score	Model category	Engraftment site	Reason
**Very high**	PDX	subcutaneous/orthotopic	best mimicking of human carcinogenesis with inter-patient and intra-tumor heterogeneity (reference mouse model)
**High**	CDX	orthotopic	orthotopic tumor growth from human cell lines in the physiologic milieu
	GEMM	not needed	immunocompetent host with temporal and spatial tumor control
**Moderate**	CDX	subcutaneous	immunodeficient host with limited genetic heterogeneity and non physiologic murine peritumoral milieu
**Low**	syngeneic	subcutaneous/orthotopic	fully murine system with poor representation of human disease and lack of tumor heterogeneity

**Table 2:** “Bench-to-bedside” scoring system.

Score	Questions (Q1-Q5)	Definition	Examples
**Very high**	++++	highly clinical relevance	A deep learning MRI-based method predicts intratumoral hypoxia before and during therapy in a PDX sarcoma mouse model, allowing the monitoring of therapy response and enabling the adjustment of schedules to prevent the emergence of resistance (15)
**High**	+++	direct translational impact	Plasma circulating tumor DNA ia a promising minimally invasive biomarker for monitoring disease burden and treatment response in RMS patients, as evidenced by its detection in both RMS mouse models and human patients (16)
**Moderate**	++	distant translation possible	Thermal drug applications via magnetic resonance-guided high-intensity focused ultrasound in an immunocompetent, syngeneic RMS mouse model (17)
**Low**	+	no translational impact expectable	Gut microbiota in RMS-bearing adiponectin knockout mice exhibits specific changes, such as decreased abundance of *Bacteroides* and an increased abundance of *Prevotella* and *Helicobacter (* 18)

Q1: How applicable are the study findings to real-world clinical scenarios?

Q2: Does the study directly address clinical questions or problems?

Q3: How likely are the study findings to be translated into clinical applications?

Q4: Does the study offer practical solutions or tools for clinicians?

Q5: How likely are the findings to be incorporated into clinical practice?

"+" indicates poor; "++" indicates average; "+++" indicates good; "++++" indicates excellent.

## Results

**Figure 1:**
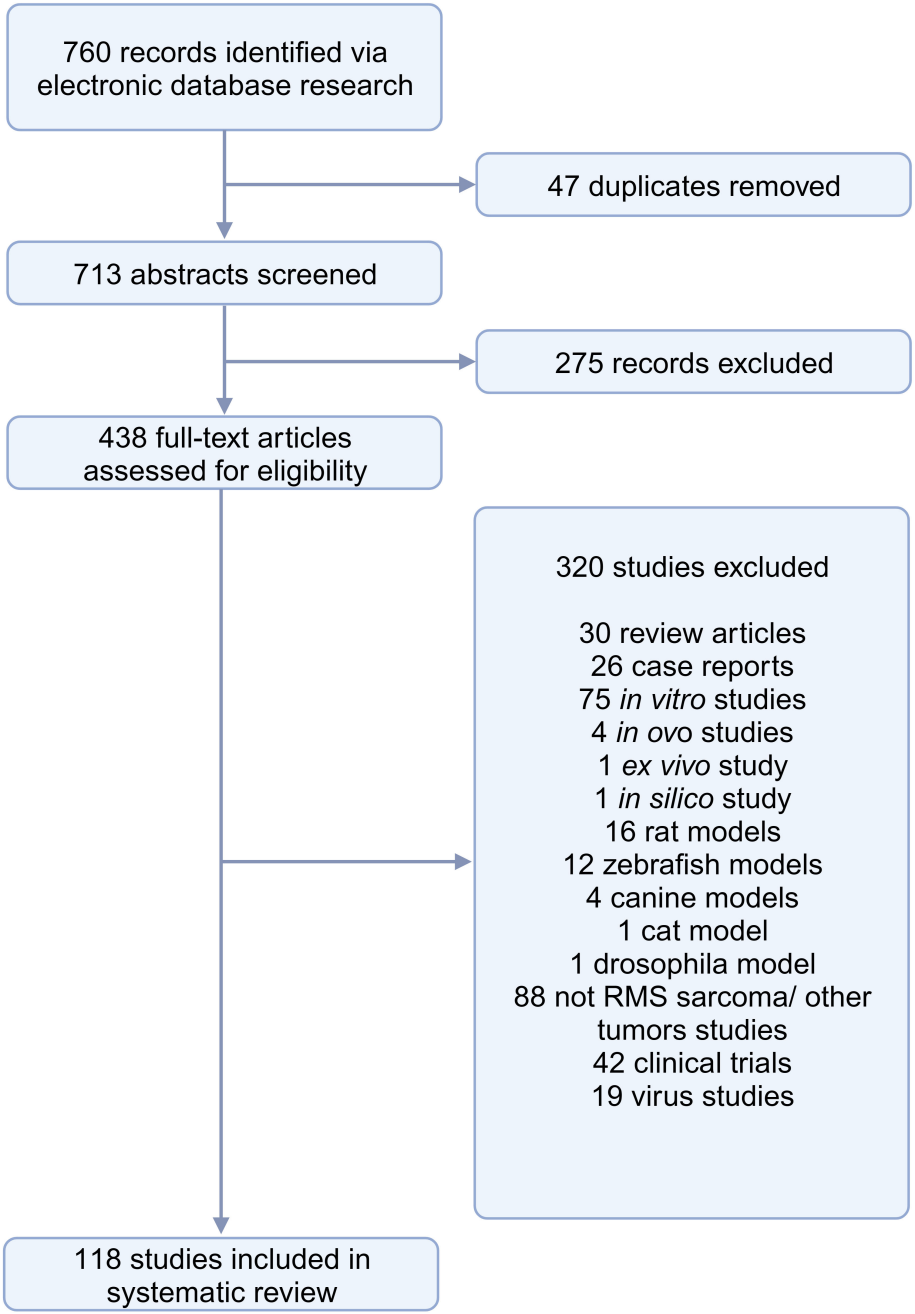
Study selection.

**Figure 2:**
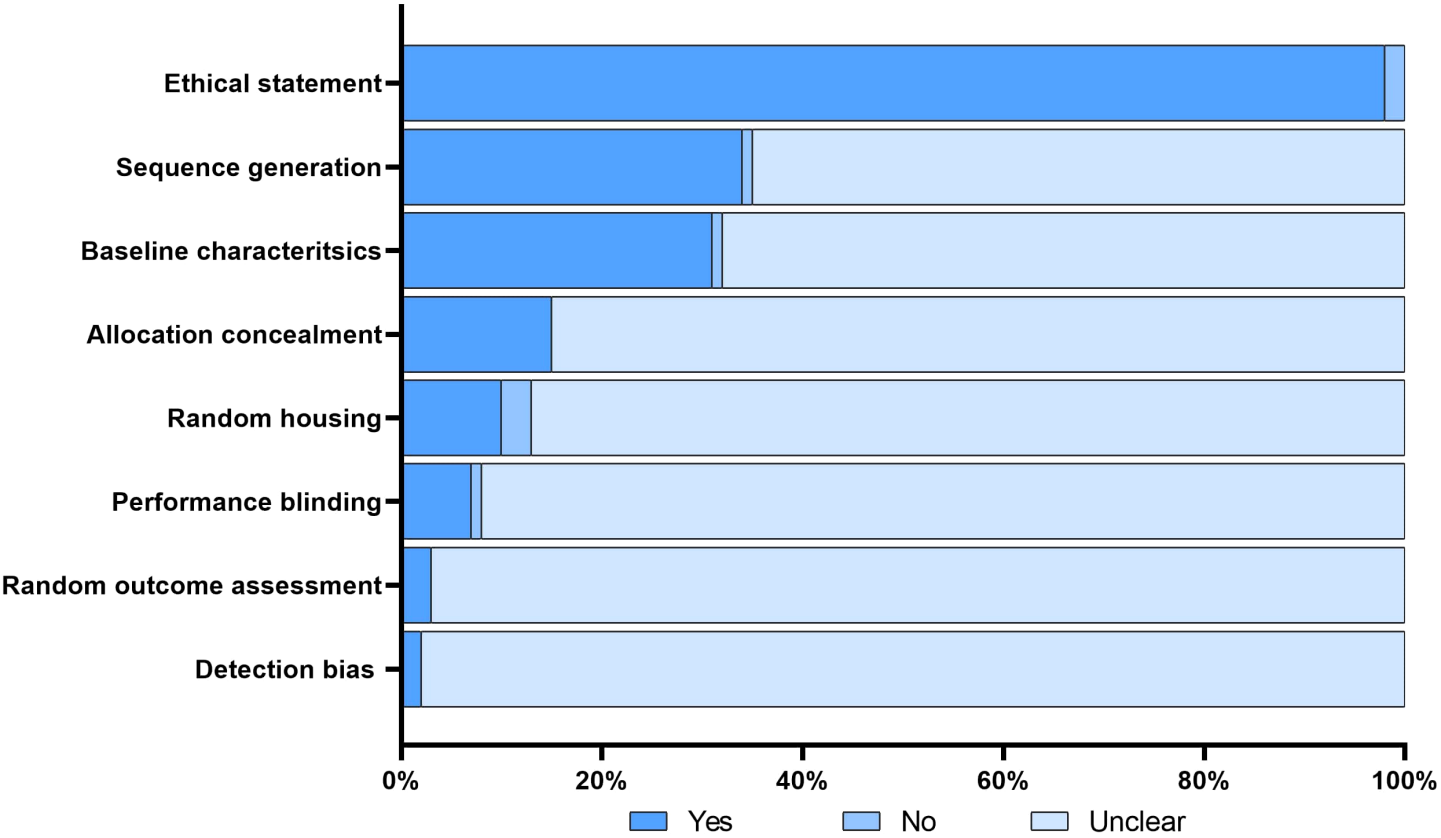
Risk of bias. The figure illustrates the risk of bias assessment for each included study using the modified SYRCLE tool. A ‘yes’ score indicates a low risk of bias; a ‘no’ score indicates a high risk of bias; and a ‘unclear’ score indicates an unknown risk of bias.

**Figure 3:**
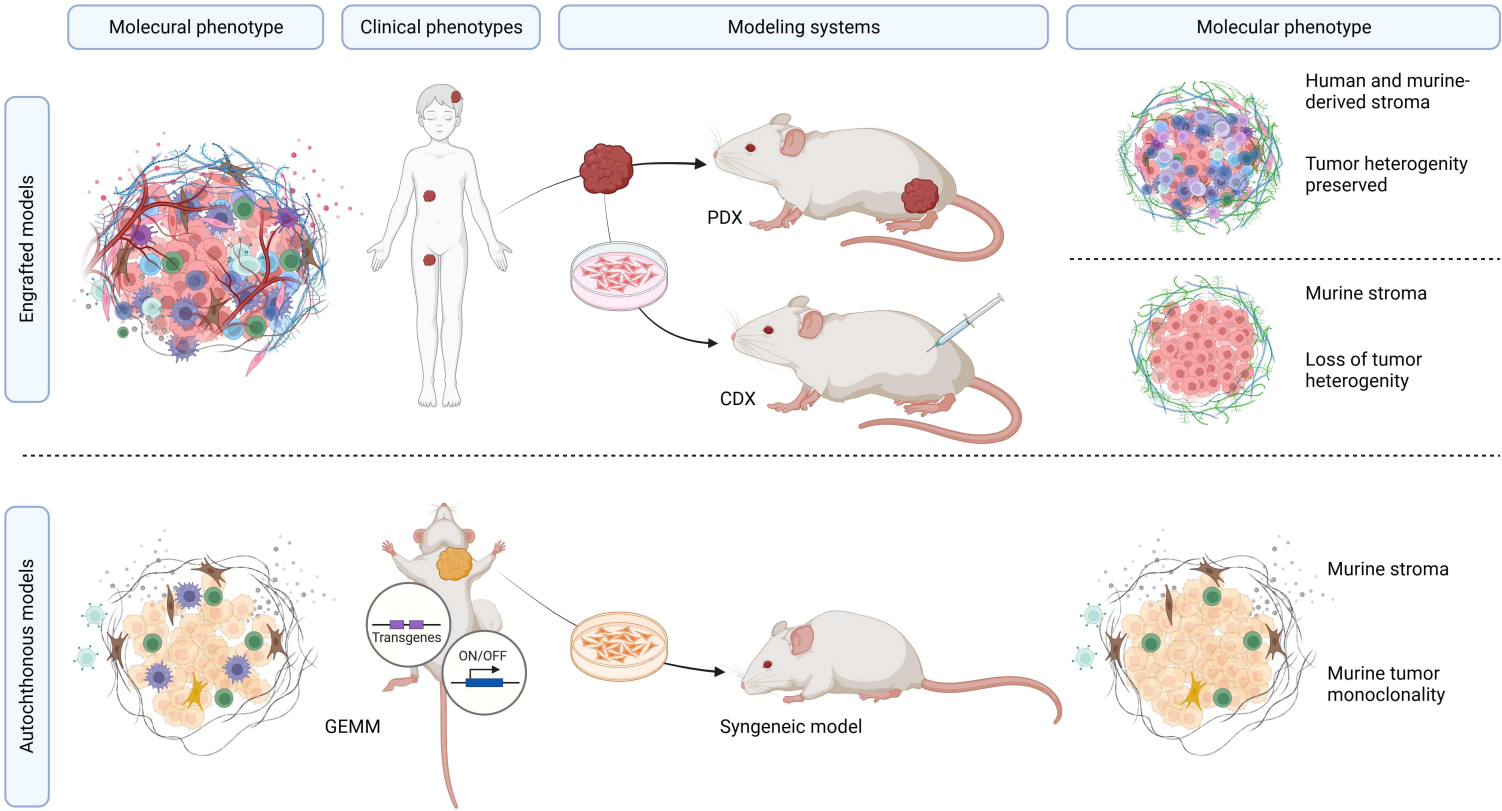
Overview of mouse model systems utilized in RMS research. Summary of preclinical RMS mouse models. The molecular tumor phenotype, in the context of the clinicopathological RMS characteristics, determines the unique features of these tumors, which can be recapitulated in both PDX and CDX models. PDX models offer the advantage of precisely mimicking the molecular, genetic, and histopathological features of the tumors while maintaining inter-patient and intra-tumor heterogeneity. CDX models, although relatively straightforward to establish and monitor, are hindered by the limited genetic diversity of human RMS cell lines within murine peritumoral microenvironments. GEMM and syngeneic models represent pure murine tumor systems with murine tumors that do not occur in humans.

The search conducted in PubMed and Web of Science yielded a total of 713 unique papers after removing 47 duplicates. After title and abstract screening, 275 papers were excluded, leaving 438 articles (**Figure 1**). Among these, 118 articles (26.9%) met the eligibility criteria and were considered for qualitative synthesis. **Supplementary Table 1**. contains the comprehensive list of included studies. **Supplementary Figure 1**. illustrates the geographical coverage of the data, encompassing 19 countries across 4 continents. The results of the risk of bias within the included studies are reported in **Figure 2**. Besides providing information on ethical statements, the majority of studies offered insufficient information regarding selection bias (sequence generation, baseline characteristics, and allocation concealment) and performance bias (random housing and investigator blinding). The mean sample size of animals per study was 65.1 ± 107.8, with a range from 3 to 499 (data available for n = 21 studies, 17.8%), and the mean sample size per treatment group was 7.9 ± 2.6, with a range from 4 to 12 (data available for n = 26 studies, 22.1%). Among different RMS mouse systems utilized in our study sample (n = 118), cell line-derived xenografts (CDX) emerged as the most commonly used (n = 75, 63.6%), followed by patient-derived xenografts (PDX) and syngeneic models, each accounting for 11.9% (n = 14). A smaller subset of studies utilized exclusively genetically engineered mouse models (GEMM) (n = 7, 5.9%). There were also combinations of different model categories, such as PDX/CDX or GEMM/syngeneic, accounting for 5.9% (n = 7). Additionally, one study employed a virus-induced RMS model, representing 0.8% of the total studies. The summary of various existing mouse model systems utilized in RMS research is presented in **Figure 3**.

Different histological CDX subtypes were established by using different human cell lines. Among these, 40.0% (n = 30) of articles represented aRMS or FP subtypes, including RH30, RH10, RH5, and SJCHR30 cell lines. Furthermore, 29 (38.7%) studies represented embryonal rhabdomyosarcoma (eRMS) or fusion-negative (FN) subtypes, such as RD, NSTS-11, and RH36 cell lines. Fifteen (20.0%) studies involved a combination of both aRMS and eRMS subtypes. In one study (1.3%) the RMS phenotype was spindle cell/sclerosing. Among studies utilizing syngeneic mouse models, nine (64.3%) used the M3-9-M cell lines, which represents the eRMS phenotype.

Among all CDX, PDX, and syngeneic models, 28.8% (n = 34) represented aRMS/FP phenotypes and 38.1% (n = 45) were eRMS/FN phenotypes. Furthermore, 22.0% (n = 26) used a combination of aRMS and eRMS phenotypes. Lastly, 11.0% (n = 13) were attributed to various non-aRMS/non-eRMS phenotypes, including ssRMS, or cases that were not classified or had undefined phenotypes.

The most common anatomical site for cell inoculation in CDX models or tumor tissue transplantation in PDX models was the subcutaneous tissue (ectopic model) (n = 66, 55.9%) followed by muscle tissue (orthotopic model) (n = 29, 24.6%). In a subset of studies (n = 7, 5.9%), the combination of different engraftment sites, such as ectopic/orthotopic or orthotopic/intravenous, was described. In 8.5% (n = 10) of the studies, no allo- or xenotransplantation was performed.

**Figure 4:**
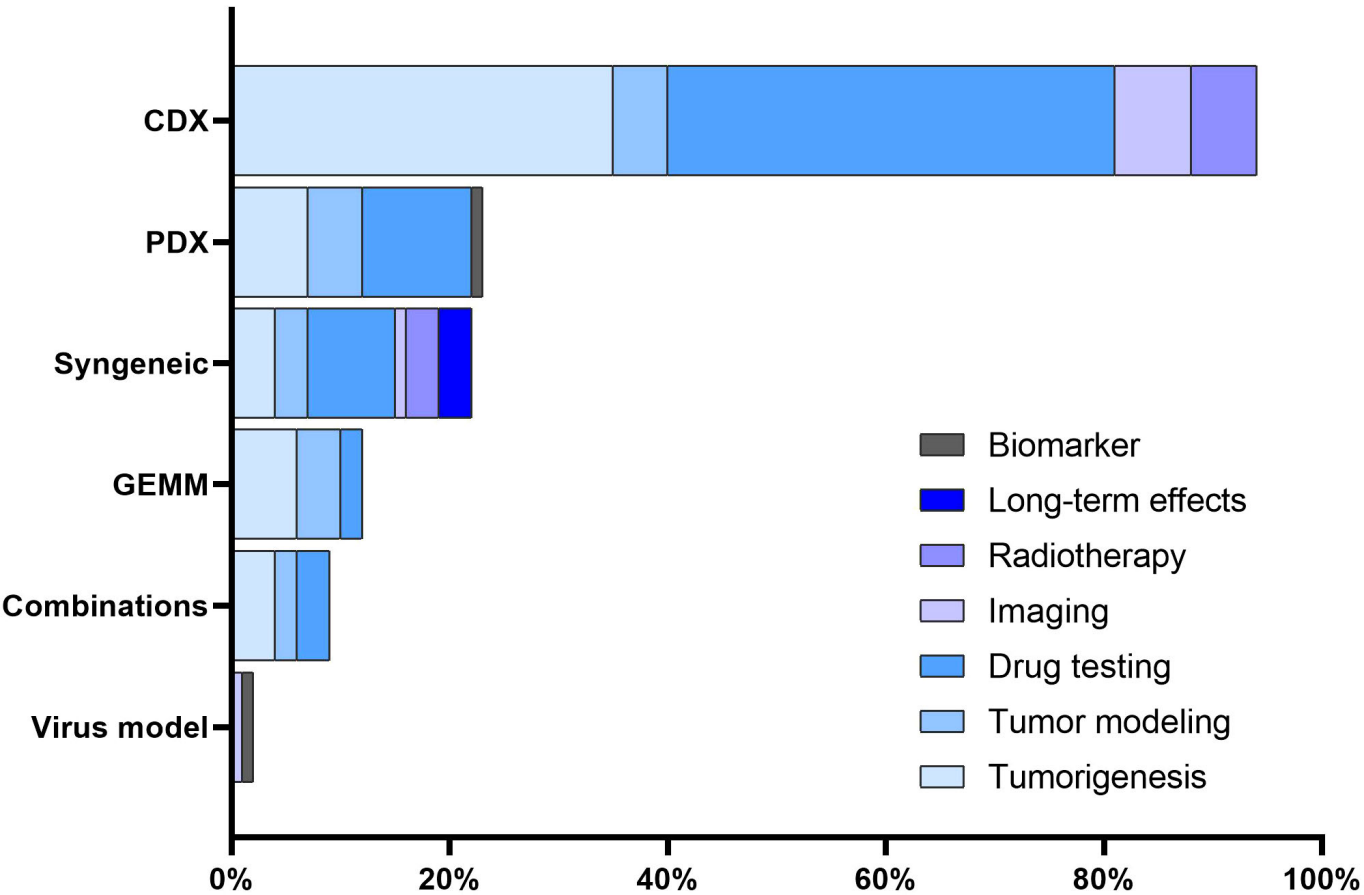
Association between mouse models and utilization areas. The distribution of various mouse RMS models across different utilization areas.

The majority of studies focused on assessing the efficacy of novel therapeutic agents or drug combinations (n = 64, 54.2%). Tumorigenesis emerged as another prominent area of interest (n = 56, 47.5%). A subset of the studies (n = 19, 16.1%) was devoted to tumor modeling. Further, RMS models were used for testing imaging techniques, as observed in 7.6% (n = 9) of the studies. Six studies (5.1%) delved into investigating radiotherapy. Lastly, a limited number of studies (n = 3, 2.5%) examined the long-term effects related to radiochemotherapy and the development and validation of biomarkers (n = 1, 0.8%). Notably, no studies in our 5-year cohort sample assessed radiation techniques or evaluated surgery-related outcomes, such as the assessment of surgical resection or testing of intraoperative imaging techniques. The association between mouse model categories and their utilization areas is presented in **Figure 4**.

Studies investigating local tumor growth were more prevalent (n = 102, 86.4%) compared to studies modeling metastatic outgrowth (n = 16, 13.6%). Among studies of tumor dissemination, 68.7% (n = 11) were experimentally induced, either by intravenous cell application (tail model, n = 8, 68.7%) or by intraperitoneal injection (n = 3, 31.3%). The remaining five studies (31.3%) represented spontaneous metastatic models (GEMM, CDX, and syngeneic models).

Next, we assigned scores to the mouse models according to their capacity to reproduce human tumors. A significant portion of the preclinical models (n = 54, 45.8%) received a ‘moderate’ score. Orthotopic CDX models and GEMM which scored highly accounted for 24.6% (n = 29). Two GEMM models were down-classified to the ‘low’ category, with one study reporting the accidental development of RMS in an ovarian cancer mouse model due to non-specific genetic manipulation (19), and the other study reporting IGF-2 overexpressing pelvic RMS with concomitant salivary carcinoma (20). Ectopic or orthotopic PDX models accounted for 15.3% (n = 18) of trials. The minority of the studies (n = 17, 14.4%) were scored as ‘low’.

**Figure 5:**
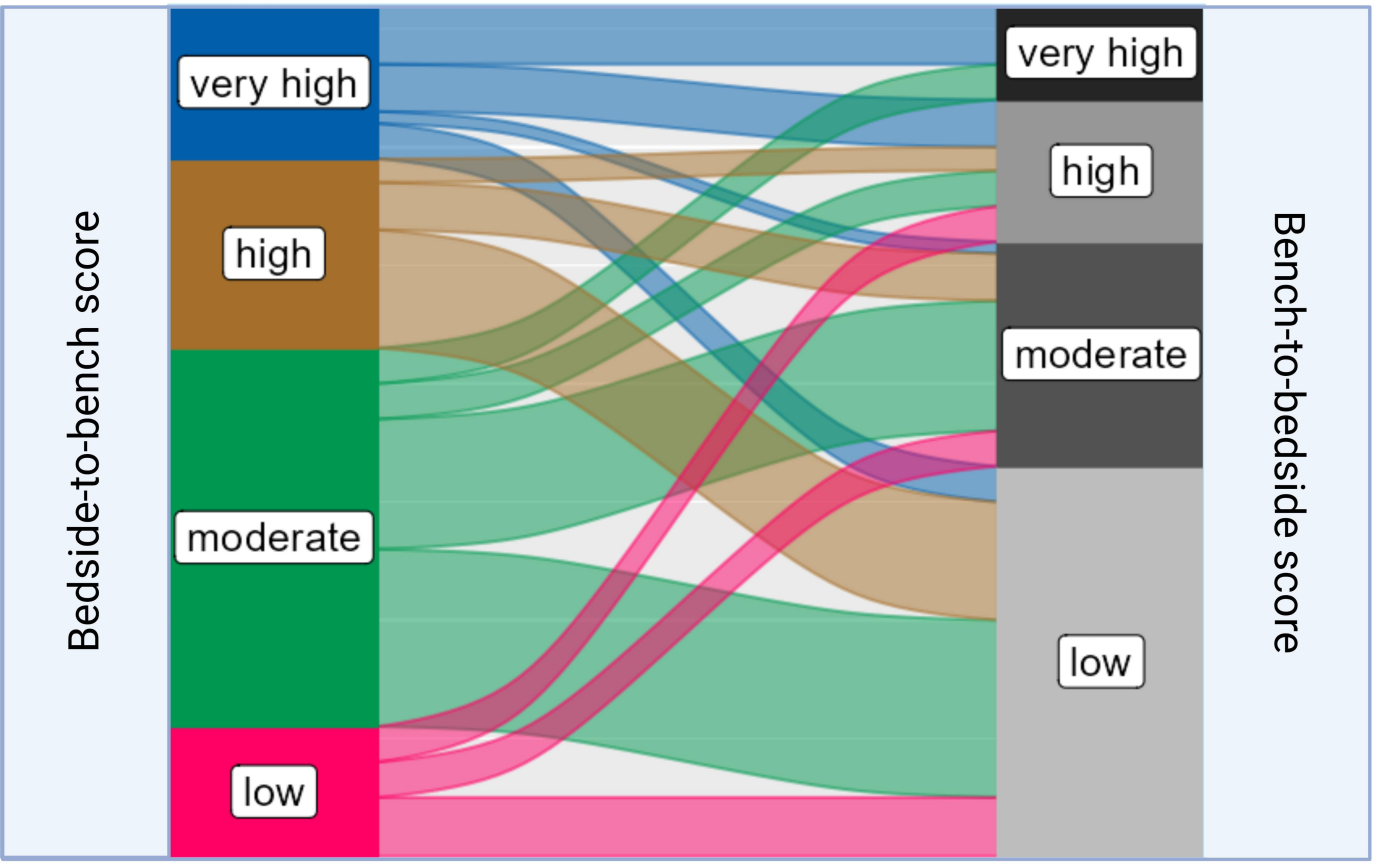
Sankey plot linking the distribution of different “bedside-to-bench” and “bench-to-bedside” scores within RMS mouse models. The Sankey plot visualizes the relationship between Bedside-to-Bench and Bench-to-Bedside scores. The width of the flowing paths represents the relative frequency of models falling within specific score ranges. The Bedside-to-Bench Score gauges the fidelity of tumor recapitulation in mouse models, while the Bench-to-Bedside Score evaluates the translational relevance of these models.

To access the translational relevance, we excluded studies on basic oncological research (n = 46, 39.0%), as they would be *a priori* scored as “low”. Among the remaining 72 studies (61.0%), 45.8% (n=33) were scored as “low”, 26.4% (n = 19) as “moderate”, 16.7% (n = 12) as “high”, and 11.1% (n=8) as “very high”. Studies with “very high” translational impact used Single Mouse Testing protocols, which enabled an evaluation of the sensitivity of RMS xenografts to chemotherapy treatment (21). Another PDX model that scored “very high” developed a multi-OMICS pipeline to analyze PDXs by integrating patient treatment history involving molecular data (22). It is important to note that preclinical models, which were scored as “high” or “very high” in terms of tumor mimicking did not necessarily correspond with “high” or “very high” translational scores. Similarly, preclinical mouse systems with low translational impact were not always graded to a “low” phenotypisation level, as demonstrated by the Sankey plot (**Figure 5**).

## Discussion

### Mouse model systems for RMS

Overall, five core mouse model systems are commonly employed in preclinical cancer research: syngeneic, CDX, GEMM, PDX, and environmental models. Notably, all of these models, except for environmental models, have found application in recent RMS studies. The distribution of RMS mouse models (CDX: 63.6%; PDX: 11.9%; syngeneic: 11.9%; GEMM: 5.9%; combination models: 5.9%) observed in our study differed from the distribution reported in a systematic survey of the literature that explored the utilization of mouse models in various solid tumors (CDX: 82%, GEMM: 24%, PDX: 7%, environmentally induced models: 6%) (23). Specifically, GEMM and environmentally induced models were underrepresented in our study, while PDX models were more prevalent.

Syngeneic models are created by implanting tumor cells from a genetically identical mouse into recipient mice of the same strain. Due to the shared genetic background between the tumor and the immune system, syngeneic models mirror the murine tumor microenvironment, enabling the exploration of immune interactions, tumor immunity, and responses to immunotherapy. One of the examples within the literature we reviewed is the study on the M3-9-M syngeneic orthotopic tumor model, which spontaneously metastasizes to the lungs, allowing a detailed evaluation of the (pre)metastatic niche (24). The M3-9-M cell line is derived from an RMS occurring in C57BL/6 mice transgenic for HGF with p53 mutation (25, 26). Similarly, *Nakahata et al.* developed a GEMM for RMS through MyoD-Cre-mediated introduction of mutant *K-RasG12D* with perturbations in p53. The resulting tumor cell lines were orthotopically implanted in immunocompetent mice (GEM-Derived allograft), exhibiting histological features comparable to the primary tumors (27). However, it is crucial to consider that these RMSs remain exclusively murine tumors that do not naturally occur in humans, which significantly hamper the clinical translationality of these models.

RMS modeling by xenotransplantation of human tumor cells into immunocompromised mice accounted for 63.6% of the cases in our survey. Despite limitations of CDX models, including the lack of immune interactions, low intra-tumor heterogeneity, and the lack the complex tumor microenvironment, the frequent utilization of such models can be attributed to their widespread availability, ease of cell culturing, high throughput capabilities, cost-effectiveness, and time efficiency. Among analyzed studies, the most commonly used tumor cell lines were RH30, RH10, RH5, and SJCHR30, representing aRMS/FP-RMS phenotypes, and RD, NSTS-11, and RH36 cell lines, representing eRMS/FN-RMS.

Of interest, the RH30 cell line is derived from the bone marrow metastasis of a 16-year-old male of untreated aRMS (28). In our sample, this cell line was used to establish different RMS phenotypes including peritoneal sarcomatosis (29), orthotopicCDX (30), ectopicCDX (31), or metastatic CDX model by intravenous cell injection (32). The question arises as to whether cell cultures derived from metastatic origins can accurately represent the behavior of the primary tumor in a mouse model. Metastatic cells undergo a series of changes that enable them to detach from the primary tumor, invade surrounding tissues, and enter the bloodstream or lymphatic system (33). Consequently, the microenvironment and genetic characteristics of metastatic cells differ from those of the primary tumor. The RH10 cell line is derived from a perineal relapse of a 15-year-old female who had previously undergone extensive treatment of the primary tumor with vincristine, cyclophosphamide, dactinomycin, and doxorubicin (34). Recent preclinical studies within our analyzed cohort utilized the RH10 cell line in ectopic CDX models to evaluate the efficacy of chemotherapeutics (35, 36). Importantly, relapsed tumors significantly differ from primary tumors due to the acquisition of new genetic mutations, changes in tumor microenvironment, treatment resistance mechanisms, and adaptation to different growth conditions (37–39). Another frequently used cell line was RD, derived from a pelvic mass of a 7-year-old female who had undergone previous treatment using cyclophosphamide and radiation, established in 1969 (40). In our study, RD cells were used to establish ectopic (41–43) and orthotopic (44) xenografts. Importantly, previous chemotherapy and radiotherapy may lead to multiple changes in tumor biology, which is different from the biology of the primary tumor including genetic mutations and alterations in the DNA and changes in tumor heterogeneity (45, 46). GEMM models of RMS were infrequently utilized (n = 10), which may be attributed to their technical complexity, long development times, and high cost. PDX models were the second most commonly used tumor model system in our study (n = 14, 11.9%). Since PDX models retain the molecular, genetic, and histological features of the human tumor, they are the only preclinical systems mimicking inter-patient and intra-tumor heterogeneity, albeit in immunocompromised organisms.

Both syngeneic, CDX, and PDX models can be established through ectopic subcutaneous or orthotopic intramuscular cell/tissue transplantation. Of note, the subcutaneous tissue lacks the native microenvironment of the original tumor site, significantly affecting tumor behavior and interactions with surrounding tissues, making dissemination less likely. In contrast, orthotopic cell injection or tumor tissue implantation enables tumor growth in a more similar microenvironment, facilitating studies on invasion, metastasis, and interactions with surrounding tissues (47). Although orthotopic models are considered superior to subcutaneous models, the majority of studies analyzed in this review utilized ectopic models (n = 66, 55.9%). This may be attributed to the fact that subcutaneous tumors offer easier accessibility for tumor monitoring and manipulation, involve simplified surgical procedures, and result in quicker tumor establishment compared to orthotopic models.

Although orthotopic PDX models have been recognized as the closest mimicry of human RMS, there are some limitations within this model system. One of these is the lack of a functional immune system, which eliminates critical interactions between the tumor and immune cells within the tumor microenvironment. As such, the understanding of these interactions is crucial, given that the immune checkpoint axis and peritumoral immune cells are regarded as potent therapeutic targets (48). Consequently, children in advanced stages of RMS, in whom conventional treatments are ineffective, may benefit from immunotherapeutic treatment modalities. Since conducting preclinical immunotherapeutic studies on human RMS tumors using PDX models is not achievable, the use of animals with a human-adapted immune system becomes necessary. The first RMS xenograft model using human-adapted mice was established by our group in 2010 (49). The protocol included sublethal irradiation of NOD/LtSz-scid IL2rγ^null^-mice, transplantation of human CD34+-cells, and subcutaneous xenotransplantation of human alveolar and embryonal RMS cell lines. This model provides the opportunity to explore novel immunotherapeutic approaches in RMS. Surprisingly, when tracking citations since the first description of the humanized RMS mouse model over the past 10 years, no subsequent studies have been utilized in RMS research. When exploring the utilization of comparable humanized mouse models in other pediatric solid tumors, there is also a paucity of preclinical studies applying this animal model system, with some rare examples in neuroblastoma (50) or Ewing sarcoma (51). This might be caused by the fact that such models are technically extremely challenging and therefore reproducible experiments are difficult to achieve.

Another drawback of PDX models is that during serial tumor transplantation, there is a probability of tumor changes with each subsequent passage, which affects the predictive value of the model. Thus, critical interpretation of data generated by PDX models is pivotal, as it has been shown that therapeutic studies are most predictive in low-passage models, due to the better preservation of human stromal components in the initial passages (52, 53). **Table 3** summarizes different mouse models utilized for RMS, along with their strengths, limitations, and application areas.

**Table 3:** Mouse models in RMS research: benefits, built-in limitations, and utilization areas.

Mouse category	Benefits	Built-in limitations	Purposive utilization areas
**Syngeneic**	• immunocompetent host • low cost • ease of implementation • preserved stroma–cancer cell interactivity	• fully murine system with poor representation of human disease • lack of heterogeneity • rapid non-autochthonous growth	• testing immunotherapeutic approaches • investigation of tumor-microenvironment interactions
**CDX**	• ease of implementation • easy access to follow tumor growth and monitor response to treatment (subcutaneous xenografts) • commercially available • high-throughput • reproducibility	• immunodeficient host • limited genetic heterogeneity • murine peritumoral milieu • surgical skills (orthotopic models) • lack of heterogeneity	• preliminary evaluation of potential therapeutic agents • biomarker discovery • tumorigenesis
**GEMM**	• temporal and spatial control • immunocompetent host	• time consuming • cost • fully murine system	• tumorigenesis • immune therapies
**PDX**	• best mimicking of human carcinogenesis (molecular, genetic, and histopathological features of the originating tumors are preserved over limited passages of *in vivo* expansion) • inter-patient and intra-tumor heterogeneity	• immunodeficient host • murine peritumoral milieu (human stroma is only initially present) • genetic and phenotypic alteration of transplant in host • surgical skills (orthotopic models) • low implantation rate (especially in eRMS phenotypes)	• tumorigenesis • exploring the pharmacokinetics and pharmacodynamics • drug resistance mechanisms

### From bedside to bench

Outcomes of patients with eRMS significantly differ from those with FP-aRMS. While the eRMS subtype displays favorable histology with 5-year survival rates of 70-80% in the absence of disseminated disease (54), the FP-RMS subtype with frequently occurred chromosomal translocations is associated with a poor prognosis resulting in a 3-year overall survival rate of 34% (55). In our systematic literature survey, we found that the majority of preclinical RMS studies accurately reported the histological RMS phenotype, even in the pure murine RMS tumors (syngeneic, GEMM, based on the histological tumor appearance), with 38.1% of the studies reproducing eRMS/FN-RMS phenotypes, 28.8% representing aRMS/FP-RMS, 22% depicting a combination of aRMS/eRMS phenotypes, and 11% utilizing non-aRMS/non-eRMS phenotypes. Although mixed aRMS/eRMS patient phenotypes are clinically possible (56, 57), preclinical studies in our sample used aRMS and eRMS separately. *Ghilu et al.* assessed drug sensitivity and resistance in different aRMS and eRMS CDX and PDX models using Single Mouse Testing protocol (58). Another example is the study conducted by *Stewart et al.*, utilizing the orthotopic PDX model of both aRMS and eRMS to test therapeutic targets for RMS through genomic, epigenomic, and proteomic analyses (59).

Primary anatomic tumor sites have different locoregional lymph node involvement and therefore might impact the prognosis, with reported survival at 5 years of ~90% for genitourinary (excluding bladder/prostate) RMS (60), ~85% for localized bladder/prostate (61), ~80% for head/neck (non-parameningeal) RMS (60), ~ 70% for parameningeal (62) and ~65% for RMS affecting extremity (63). In analyzed preclinical trials, orthotopic mouse models (syngeneic, CDX, PDX) mimicked most likely extremity RMS phenotypes. No PDX or syngeneic models mimicked genitourinary or head/neck RMS phenotypes. Moreover, ectopic mouse models did not recapitulate any of the human anatomical tumor sites, thus having very limited translational value. While orthotopic mouse models recapitulated the limb RMS phenotype, a variety of additional tumor locations were observed merely in GEMM systems within our study cohort, including the abdomen, back, head/neck, rib cage, pelvis, body wall, and tongue (19, 20, 27, 64–70).

While patients with nonmetastatic localized RMS tumors have good to excellent prognoses depending on tumor site/size, age, and *FOXO1* fusion status, children with disseminated disease have a poor prognosis with OS ~ 30% (55). Metastatic disease modeling was carried out in a minority of the included studies (13.6%). Among them, 37.5% of studies were able to induce metastasis spontaneously using GEMM (20, 27), orthotopic CDX (71, 72) or even ectopic xenografts (73), all of which disseminated to the lungs. Another approach to inducing metastases was through the injection of tumor cells, either intravenously (tail vein murine models), resulting in lung (24, 74–78), bone marrow (32), and liver/spleen (79) metastasis, or intraperitoneally, resulting in peritoneal sarcomatosis (xenograft models with intraperitoneal dissemination) (29, 80–82).

### Patient´s age

There are three prognostically relevant age groups, including children younger than 1 year with the 5-year failure-free survival (FFS) and OS of 57%/76%, children older than 1 year and younger than 10 years with FFS/OS of 80%/87%, and children older than 10 years with FFS/OS of 70%/76% (83, 84). Importantly, the poor prognosis for infants may not only be determined by intrinsic tumor factors but also by treatment modifications including lower chemotherapy doses due to higher rates of chemotherapy-related toxicity (85). In our study sample, there was only one investigation addressing mouse age on tumor development. *Ragab el*. showed that induction of the *oncNRAS* mutation in 2-week-old Ptch+/-NRasfl/+ mice (GEMM) accelerates eRMS formation compared to 4-week-old mice (68). The other studies utilized mice of different ages, including 3-week-old mice (86), mice between 4 to 8 weeks (87, 88), 6 to 8 weeks (41, 89), or 8 to 12 weeks (90, 91), irrespective of the mouse model subtype. It would be reasonable to correlate patient age groups (<1 year, 1-9 years, and > 10 years) with mouse ages to accurately mimic age-related clinical phenotypes. Taking into account the disparate lifespans of laboratory mice (~24 months) and humans (~80 years), *Dutta et al.* described the relationship between mice and humans at all stages of growth and formulated mathematical formulas for the calculation of mice/human ages (92). Hence, replicating infant RMS would be most suitable using 0 to 3 or 4 weeks-old mice during the sucking period. Given that mice typically reach puberty around 6–10 weeks of age (while humans reach puberty at about 11.5 years), the optimal age range for simulating patients aged 1-9 years would be between 4 and 6 weeks during the juvenile period of the mouse. As mice reach adulthood at 8-12 weeks (humans generally reach adulthood at the age of 20 years), the time frame between 6-8 week-old mice would represent children > 10 years old. The adolescent RMS phenotype would be most accurately replicated in mice that are older than 10 weeks.

### From bench to bedside

Generally, preclinical RMS research focuses on two main aspects, involving critical biological and clinical inquiries (93). Open biological questions include elucidating the mechanisms facilitating invasion and metastasis in fusion positive and negative RMSs, defining the role of immune cells in the tumor microenvironment, and identifying the most predictive preclinical models for RMS. Clinically relevant questions are the exploration of immunotherapeutic treatment possibilities, the determination of combinations of targeted agents, and the improvement of local control in regions like the abdomen and pelvis. Studies included in our review addressed both biological questions, such as tumorigenesis, and clinically relevant questions, such as testing new drugs, investigating novel imaging techniques, discovering biomarkers, and reducing side effects and long-term effects.

### Tumorigenesis

In our study sample, 47.5% of all studies focused on tumorigenesis, including both FP-RMS and FN-RMS phenotypes. Among them, a minority of studies investigated mechanisms associated with tumor dissemination. So, Skrzypek et al. investigated the role of the SNAIL-dependent miRNAs miR-28-3p and miR-193a-5p in RMS development and metastasiogenesis in both RH30-ectopic xenografts and intravenous injection model (32). Another study conducted by Almacellas-Rabaiget et al. examined the role of lysyl oxidase-like 2 (LOXL2), a regulator of tumor progression and metastasis, in orthotopic xenograft aRMS models that developed spontaneous lung metastasis attributed to LOXL2 overexpression (72). In one further study in which an experimental tail vein metastasis RMS mouse model was used, Navarro et al. showed that Integrin alpha9 was essential for tumor dissemination, with reduction of metastatic formation by applying a synthetic peptide, RA08 (94). A preclinical study focusing on tumor microenvironment was conducted by Miyagaki et al., investigating the antitumor effects by suppressing lipid metabolism in an orthotopic xenograft mouse model (95). The question concerning the most predictive preclinical models for RMS remains unanswered, given that no single model can universally meet all experimental requirements. Probably, the most predictive preclinical RMS model is the most suitable for the specific research question. Overall, among all the mouse models analyzed in our study, orthotopic PDX models appear to be the most accurate in predicting clinical outcomes.

### Therapeutic agents

More than 50% of analyzed studies investigated a novel therapeutic agent or their combinations. The explanation for the high prevalence of therapeutic studies is that patients with dissemination or tumor relapse face a dismal prognosis, given the limited effectiveness of standard therapies. These novel chemotherapeutic agents are subsequently translated into clinical phase 1 and phase 2 trials (96–98). However, the therapy for RMS is multimodal, encompassing not only chemotherapy but also local tumor control through surgery and/or radiotherapy. Surprisingly, no preclinical studies focused on surgery. Moreover, in addition to the literature investigated during the 5-year timeline, there are a few preclinical studies on surgery in RMS. These include a study investigating tumor visualization using fluorescence laparoscopy (ICG) (99) and another study investigating the effect of CpG oligodeoxynucleotides as a potent immunomodulator after surgical resection of murine tumor (100).

### Radiotherapy

All of the preclinical investigations within our study sample that focused on the topic “radiotherapy” explored a range of radiosensitizing agents, including SNAI2 (101), MS-275 (102), romidepsin (103), CLR1404 (104), PXD-101 (105), and CLR127 (31). Such radiosensitizers enhance the effectiveness of radiotherapy by increasing the induction of DNA damage (106). This can be particularly crucial in cases of radioresistant RMS, where standard radiation therapy alone is not sufficient.

### Imaging

Imaging plays a central role in the diagnostic workup, therapy planning, follow-up process, and detection of relapses in RMS patients. In the examined literature cohort, there were 6 studies investigating imaging techniques in murine RMS tumors. Interestingly, the majority of studies have focused on correlation investigations between diffusion-weighted imaging (DWI)/intravoxel incoherent motion (IVIM) and histopathologic features of murine tumors (43, 107–109).

### Long-term effects

Only a limited number of studies (n = 3) addressed long-term effects related to the treatment of RMS. So, Collao et al. investigated the impact of resistance and endurance exercise training on muscle mass composition after chemotherapy with radiation in a murine RMS mouse model (88). Kallenbach et al. analyzed the late effects associated with radiotherapy on skeletal muscles, including sarcopenia, musculoskeletal frailty, and radiation-induced fobrosis (86). A similar study by Paris et al. showed that chemoradiation impairs myofiber hypertrophic growth in RMS mouse model (110). However, the limited number of studies addressing the long-term effects of radiotherapy or chemotherapy can be attributed to the challenges in justifying animal use according to the principles of the 3Rs in animal experimentation. This is particularly challenging when well-established standard radiotherapy/chemotherapy protocols are already in place in patient care.

### Biomarker

Only one study assessed the feasibility of plasma circulating tumor DNA to predict disease burden and treatment response using blood samples from RMS mouse models and patients (16). This shows that this field is understudied in preclinical research.

### Selection of an appropriate mouse model to study RMS

**Figure 6:**
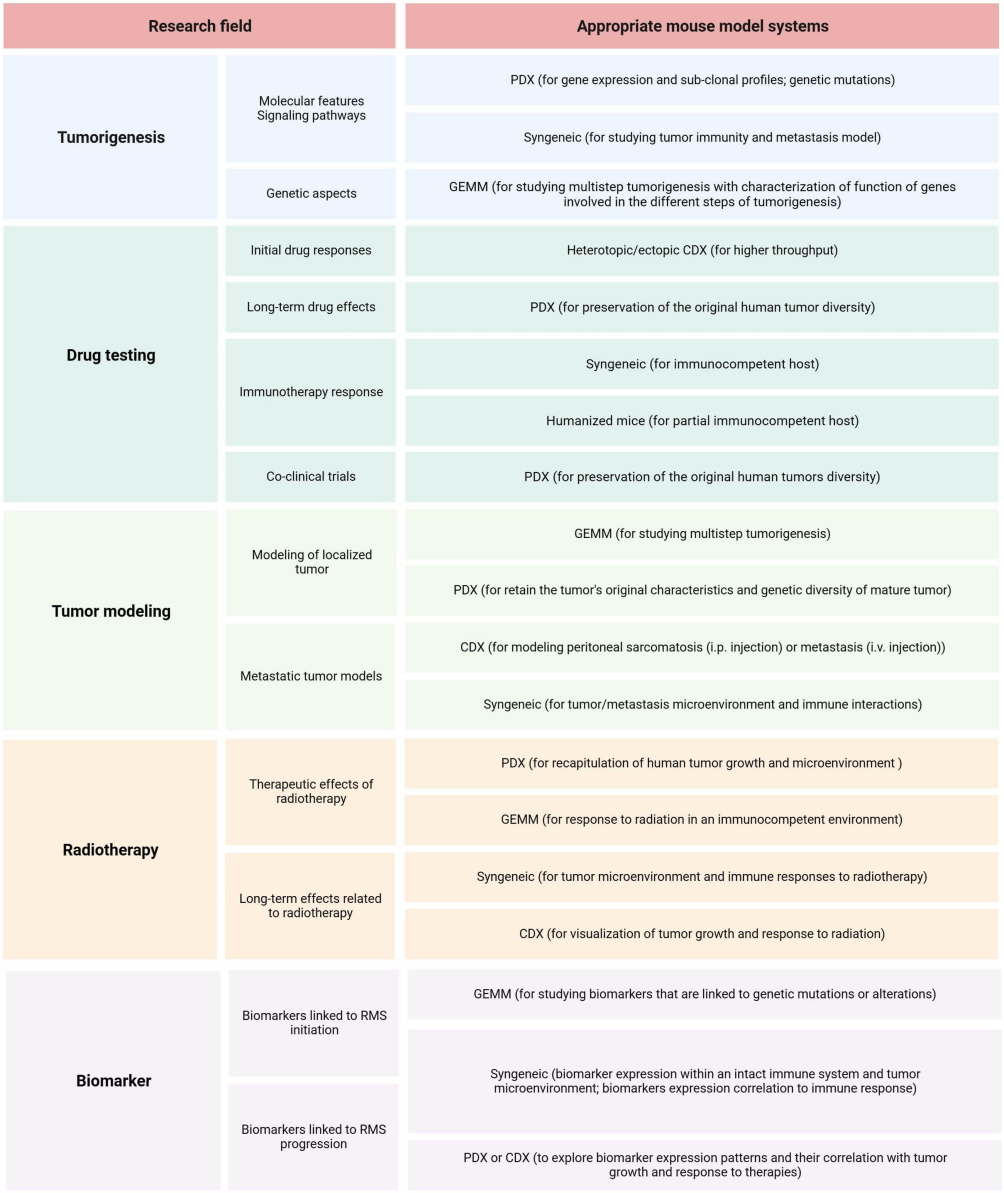
RMS mouse model selection. Summary of relevant research areas and sub-areas within preclinical RMS research, along with corresponding suggestions for mouse models.

The first step in planning preclinical experiments is selecting the most suitable model, guided by the study’s clinical and/or biological objectives. An ‘ideal’ translational model should be multifaceted, accurately reproducing the clinical situation by considering various clinicopathological and molecular-biological variables of the patient. The biological or clinical question should determine the choice of a mouse model and not the reverse. **Table 3** summarizes the strengths and limitations of core mouse models used in RMS research. Among RMS mouse models, two fundamental categories should be considered: 1) autologous (GEMM) vs. non-autologous (CDX, PDX, syngeneic) tumor models (111). The rational choice for autologous models (GEMM) includes the investigation of cancer development from *de novo* initiation stages to progression with metastatic disease within a natural immune‐proficient tumor microenvironment (112). Non-autologous transplantable tumor models (CDX or PDX) are considered for both tumorigenesis research and drug screening/validation research, as these models more precisely recapitulate human tumors (113–115). Syngeneic models are very reductionist due to the absence of human targets and should therefore only be considered for preclinical immunological and immunotherapeutic studies or as synergistic partners in combination with other more complex models such as GEMM or CDX/PDX models (116). Another fundamental categorization of RMS models includes: 1) immunocompetent (GEMM, syngeneic) and 2) immunocompromised (CDX, PDX) hosts. The rationale for selecting pure murine, yet immunocompetent hosts, is to study immunological processes within the primary tumor, tumor metastasis, and the tumor microenvironment or for immunotherapeutic drug discovery (117). It’s important to consider that curing murine tumors does not guarantee clinical success in humans even if applying more complex mouse models (118, 119). Among mouse models using immunodeficient hosts (CDX and PDX), further gradations should be made, as different mouse strains have varying levels of immunodeficiency. While athymic nude mice lack a thymus and thus have impaired T-cell-mediated immune responses (moderate level of immunodeficiency), SCID mice lack functional T- and B-cells, resulting in severe immune deficiency (120, 121). Additionally, NOD SCID mice, which are double mutants with SCID and NOD mutations, exhibit reduced natural killer (NK) cell activity (122). NSG (NOD SCID Gamma) mice are triple-mutant with SCID, NOD, and IL2rg null mutations, and they are devoid of T-, B-, and NK cells (123, 124). The success rate of establishing CDX or PDX models depends on the degree of immunodeficiency of the mouse host. SCID mice demonstrate higher engraftment efficiency compared to athymic nude mice. However, transplantation efficiencies may be lower for cells from less malignant tumors due to residual NK cells in SCID models. In contrast, NSG mice exhibit multiple deficiencies in both innate and adaptive immunity, thus making them more suitable recipients for different human solid tumor transplantation (125). When considering non-autologous CDX models, careful selection of cell application routes is essential: 1) heterotopic subcutaneous models are most suitable for initial novel drug screening studies (126), 2) orthotopic models with cell injection into gastrocnemius muscle representing a clinical phenotype of ‘extremity RMS’, and 3) metastatic models [i.v., particularly for lung metastasis or i.p. for peritoneal sarcomatosis (127, 128)]. Although certainly technically challenging to establish, both heterotopic and orthotopic PDX models should be regarded as reference mouse RMS models due to their potential to maintain human tumor architecture, intratumoral and interpatient heterogeneity, and tumor microenvironment components, enabling their multipurpose use across various application areas (113, 129–132). **Figure 6** illustrates the process of selecting the preclinical mouse model based on specific utilization areas.

In conclusion, this up-to-date review underscores the critical importance of a discerning approach in the selection and interpretation of preclinical mouse models for RMS research. The use of ectopic CDX models, even though they constitute nearly two-thirds of the research, reveals their limitations in accurately recapitulating human RMS phenotypes. Orthotopic PDX models emerge as the closest mimics of human RMS, offering high translational value. However, the absence of a functional human immune system in both CDX and PDX models hinders the exploration of critical cell interactions within the tumor microenvironment. Therefore, our paper highlights the need for further humanized mouse models that address the shortcomings of existing ones. Moving forward, research efforts should be directed towards unexplored avenues such as surgery, radiotherapy, and biomarkers.

## Data availability statement

The raw data supporting the conclusions of this article will be made available by the authors, without undue reservation.

## Author contributions

IM: Conceptualization, Data curation, Formal analysis, Investigation, Methodology, Software, Visualization, Writing – original draft, Writing – review & editing. LD: Investigation, Resources, Writing – review & editing. BW: Validation, Visualization, Writing – review & editing. PH: Investigation, Methodology, Resources, Writing – review & editing. MV: Conceptualization, Supervision, Validation, Writing – review & editing. GS: Conceptualization, Data curation, Methodology, Project administration, Resources, Supervision, Validation, Writing – review & editing.

## Supplementary material

Supplementary Figure 1 A choropleth map displays the distribution of preclinical RMS publications across 19 countries spanning four continents. The size of each circle on the map corresponds to the number of publications. The country with the highest publication count was the USA (n=57), followed by China (n=12), Italy (n=9), Spain (n=7), Japan (n=6), Switzerland (n=5), Germany (n=4), Canada (n=3), Singapore (n=3), Czech Republic (n=2), the UK (n=2), Belgium (n=1), Israel (n=1), Lebanon (n=1), Norway (n=1), Poland (n=1), Russia (n=1), South Africa (n=1), and Sweden (n=1).
