# Supplementary figures and images for: Contemporary preclinical mouse models for pediatric rhabdomyosarcoma: from bedside to bench to bedside
Source: Front Oncol. 2024 Feb 2;14:1333129. doi: 10.3389/fonc.2024.1333129 (PMC10869630; doi:10.3389/fonc.2024.1333129)

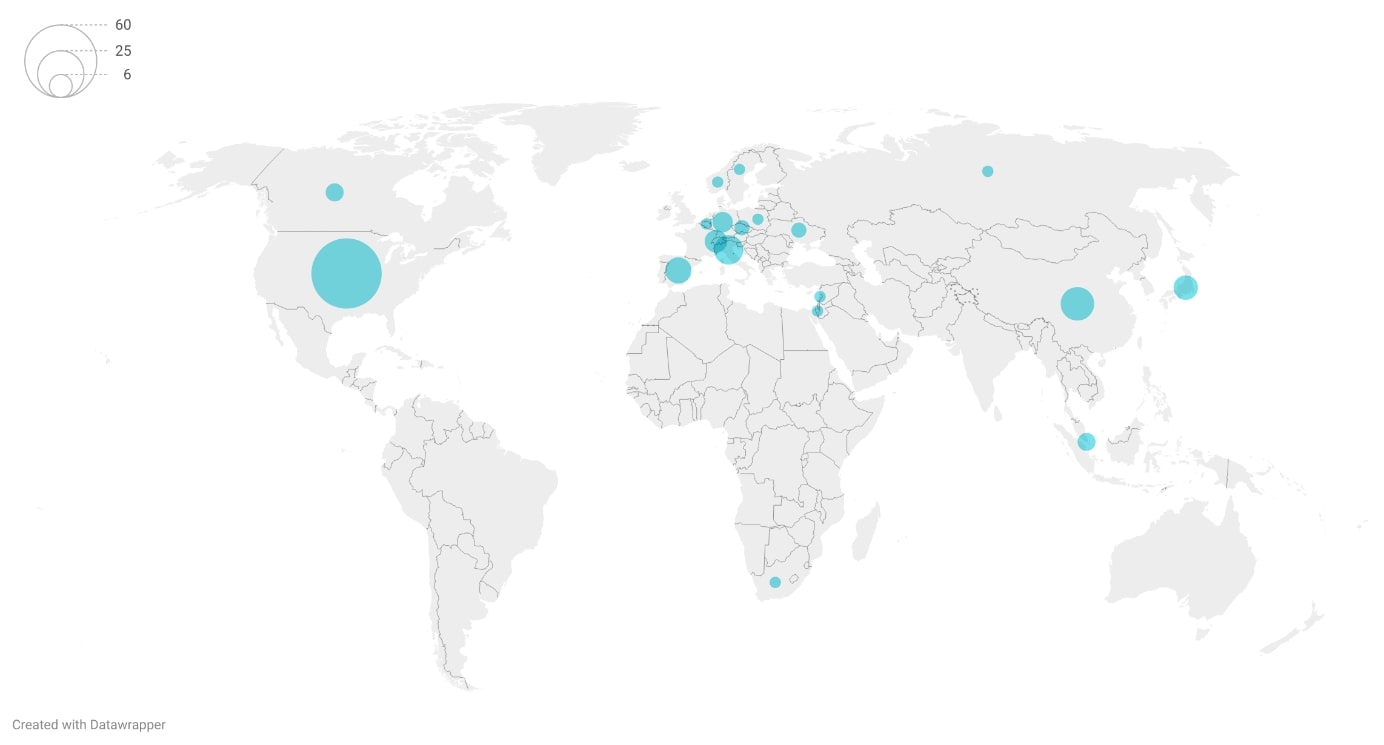

Supplement: Supplementary Figure 1 — A choropleth map displays the distribution of preclinical RMS publications across 19 countries spanning four continents. The size of each circle on the map corresponds to the number of publications. The country with the highest publication count was the USA (n=57), followed by China (n=12), Italy (n=9), Spain (n=7), Japan (n=6), Switzerland (n=5), Germany (n=4), Canada (n=3), Singapore (n=3), Czech Republic (n=2), the UK (n=2), Belgium (n=1), Israel (n=1), Lebanon (n=1), Norway (n=1), Poland (n=1), Russia (n=1), South Africa (n=1), and Sweden (n=1). [file Image_1.jpeg]
